# Meeting the Challenge of Cancer Survivorship in Public Health: Results from the Evaluation of the Chronic Disease Self-Management Program for Cancer Survivors

**DOI:** 10.3389/fpubh.2014.00214

**Published:** 2015-04-27

**Authors:** Betsy C. Risendal, Andrea Dwyer, Richard W. Seidel, Kate Lorig, Letoynia Coombs, Marcia G. Ory

**Affiliations:** ^1^Community and Behavioral Health, Colorado School of Public Health, Aurora, CO, USA; ^2^Carilion Clinic, Virginia Tech Carilion School of Medicine, Roanoke, VA, USA; ^3^School of Medicine, Stanford University, Stanford, CA, USA; ^4^Colorado Health Outcomes, University of Colorado, Aurora, CO, USA; ^5^Health Promotion and Community Health Sciences, Texas A&M Health Science Center School of Public Health, College Station, TX, USA

**Keywords:** cancer survivorship, self-management support, patient education, community-based research, effectiveness trial

## Abstract

**Introduction:**

Self-management has been identified as an important opportunity to improve health outcomes among cancer survivors. However, few evidence-based interventions are available to meet this need.

**Methods:**

The effectiveness of an adapted version of the Chronic Disease Self-Management Program for cancer survivors called Cancer Thriving and Surviving was evaluated in a randomized trial. Outcomes were assessed at baseline and 6-months post program via written survey among 244 participants in Colorado. Repeated measures analysis was used to analyze pre/post program change.

**Results:**

Statistically significant improvement was observed among those in the intervention in the following outcomes: Provider communication (+16.7% change); depression (−19.1%); energy (+13.8%); sleep (−24.9%) and stress-related problems (−19.2%); change over time was also observed in the controls for energy, sleep, and stress-related outcomes though to a lesser degree. Effect sizes of the difference in change over time observed indicate a net beneficial effect for provider communication (0.23); and decreases in depression (−0.18); pain (−0.19); problems related to stress (−0.17); and sleep (−0.20).

**Conclusion:**

Study data suggest that the self-management support from adaptation of the CDSMP can reach and appeal to cancer survivors, improves common concerns in this population, and can fill an important gap in meeting the ongoing need for management of post-diagnosis issues in this growing segment of the U.S. population.

## Introduction

The estimated lifetime risk of developing cancer is 45% among men and 38% among women, with an expected total of 1.6 million new cancer cases in 2012 (1). More individuals are living longer due to improvements in early detection and treatment, and therefore, the number of cancer survivors in the U.S. has dramatically increased. Current estimates suggest that there are over 13 million survivors alive today in the U.S., with an estimated 18 million at the end of the decade; an estimated 65% of all survivors live 5 years or more. This dramatic increase in the survivor population has consequences for both the health of survivors and the healthcare system. For example, many survivors experience late and long-term effects from cancer and its treatment. Pain, fatigue, depression, impaired physical function, and fear of recurrence are among the most common consequences of cancer as described in the landmark report by the Institute of Medicine, “From Cancer Patient to Cancer Survivor: Lost in Transition” (2). This Report also concluded that the care of cancer survivors is fragmented and poorly coordinated and that self-management support can help promote the delivery of quality care and improved health outcomes in this population. Further, cancer survivors die from non-cancer causes at a rate higher than the general population (3, 4) likely due to the long-term side effects of cancer and its treatment and risk factors common to both cancer and non-cancer causes of death.

The Chronic Care Model [CCM; (5)] is a rigorously evaluated and widely adopted approach to care management for chronic conditions and features self-management support as one of the key components for assuring quality healthcare. Self-management is defined as comprehensive engagement of the patient in problem solving, decision making, and daily health-related behaviors in partnership with their healthcare provider and community (6). A 2007 review by Nolte et al. (7) found many benefits from self-management programs are also relevant to survivorship such as: improved knowledge, acquisition of skills, symptom management, and ability to self-monitor health and healthcare needs.

The Chronic Disease Self-Management Program (CDSMP) is one of the few evidence-based interventions available across a variety of health-related conditions for comprehensive self-management support (8). While there are now specialized versions for some chronic conditions such as diabetes, chronic pain, HIV/AIDS, and arthritis^1^, an adapted version for cancer survivors has only recently been developed for use and testing in the U.S. The purpose of the current paper is to describe the findings from the 6-month outcome evaluation of cancer thriving and surviving (CTS) among over 200 cancer survivors in the post-treatment phase who participated in a randomized trial in Colorado between 2011 and 2013. In this paper, we report the effectiveness of the evidence-based CDSMP translated to cancer survivors by comparing the magnitude of the effect observed in the intervention vs. control group over time.

## Materials and Methods

### Intervention

Developed by researchers from the Stanford Patient Education Research Center at Stanford University, the model for the CDSMP program entails a series of six weekly small-group sessions led by trained facilitators. The model is based on social cognitive theory (9) to focus on building skills, sharing experiences, and support among the participants to maximize engagement. Sessions follow a standardized curriculum detailed in a program manual to promote fidelity to the following program elements: brainstorming, action plan formulation, action plan feedback, problem solving, and decision making (10).

In brief, adaptations to the CDSMP for cancer survivors were guided by the research of Foster et al. (11) and the subsequent conceptual model (12) to include restoration of self-confidence, adjustment to changed self, and confidence to self-manage cancer-related problems. The resultant CTS curriculum was initially developed by Macmillan Cancer support in the U.K and subsequently modified by the Stanford Patient Education Center to incorporate language more common to the U.S.

Researchers at the Colorado School of Public Health (CSPH) partnered with the Consortium for Older Adult Wellness (COAW) to deliver the program. COAW is a community-based agency with state-wide license to deliver the evidence-based CDSMP. Individuals who were already trained and licensed to provide the CDSMP workshops and who were also cancer survivors completed a 2-day cross-training program led by the Stanford Patient Education Center to ensure fidelity to the model.

### Recruitment

Cancer survivors throughout the “Front Range” of Colorado, where roughly two-thirds of state’s population resides, were approached in a variety of outreach methods including: interactions with cancer center staff and brochures left at medical offices, mailed to homes using mailing lists from local cancer survivor programs, distributed at cancer survivor local events, and media. Potential respondents identified from these routes were contacted by COAW personnel (located in Denver, also within the Front Range) to assess interest and eligibility for participation in the program. Participants were allocated to the intervention or control groups for the analytic evaluation. Inclusion in the program required participants to be over the age of 21 years and diagnosed with cancer that required radiation, surgical, or adjuvant chemotherapy treatment, but not to be in active treatment at the time of enrollment. Persons currently receiving maintenance therapies for cancer delivered after completion of primary treatment (such as anti-hormonal treatments) were eligible. Support persons/caregivers of the above were also allowed to attend. All persons had to speak and read/write in English, and also agree to attend in-person classes and arrange transportation to attend classes. Persons in end-of-life care or currently undergoing active treatment for cancer were excluded, as were individuals over the age of 79 years. Approval to conduct the research was obtained by the Colorado Multiple Institutional Review Board; participants provided signed informed consent. No incentives were offered to potential participants.

### Intervention delivery

Twenty-seven workshops were delivered in Colorado between August 2011 and January 2013. Each workshop consisted of six 2.5 h sessions led by two facilitators as described above. Facilitators were periodically observed by Master Trainers and provided written feedback to monitor fidelity and quality assurance.

### Data collection

Written self-administered surveys were collected from participants at baseline and for final follow-up measure (6 months after program completion). Instruments were from the Stanford CDSMP Evaluation^2^ and have been widely used in many health and aging studies (13, 14) and are viewed as pragmatic measures (15). Participants were asked at baseline to self-report demographic characteristics (age, gender, marital status, race/ethnicity) as well as cancer-related history (caregiver, time since diagnosis, type of cancer, co-morbid conditions).

### Study design

This study deployed a randomized controlled trial design, where participants were randomized in a 2:1 ratio following consent to the intervention vs. control group. Since the purpose of this study was to evaluate effectiveness rather than efficacy, we intentionally sought to maximize the number of participants receiving the intervention so that we could gain more experience to inform implementation and also to improve the generalizability of the results by broadening the characteristics and delivery while still utilizing a valid comparison group. Participants were randomized to group assignment using a random number generator by the research coordinator who was separate from the intervention delivery. Caregivers/support persons were randomized as a pair with their survivor so they could attend sessions together, and therefore, not counted toward the 2:1 ratio. Persons who consented and were randomly assigned to the control group were offered to attend the CTS workshops after the final evaluation assessment was collected at 6-months following consent; thus their data served as the control for those randomized to the intervention group. This design was chosen to facilitate retention of controls over the 6-month time period between consent and assessment of the main outcome measures at 6 months post-program (in order to mirror the CDSMP evaluation plan), and to gain more experience with intervention delivery.

### Outcomes

Our hypothesis was that the intervention would produce improvement in outcomes directly related to health beliefs and behaviors related to physical activity (days active/minutes active), self-efficacy, and communication with providers. Secondary outcomes of interest included self-reported health and symptoms (health status, depression, energy, pain, sleep, and stress).

The following describes measures employed in this study:

- Days active, minutes active: days active, minutes active: respondents were asked how many days in the past week they were physically active or exercising for at least 30 min and how many total minutes in the past week they were physically active or exercising, including brisk walking, running, dancing, bicycling, water exercise, etc., that may cause faster breathing or heartbeat, or feeling warmer. For the current analyses, we are using continuous count data for number of minutes exercised and number of days exercised. Respondents were asked how many days in past week they were physically active or exercising for at least 30 min and how many total minutes in the past week they were physically active or exercising (including brisk walking, running, dancing, bicycling, water exercise, etc.) that may cause faster breathing or heartbeat, or feeling warmer. For the current analyses, we are using continuous count data for number of minutes exercised and number of days exercised.
- Participant care seeking behaviors (communication with physicians): communication with a physician was measured using a three-item scale, which asked participants if they did the following things when visiting a physician: prepare a list of questions, ask questions about things they want to know or do not understand, and discuss personal problems. Scores for these items ranged from never (0) to always (5). If respondents answered at least two of these items, the scale was calculated as the mean of the non-missing items. Higher scores represent better communication with a physician. An increase or positive change is desirable.
- Self-efficacy: this was measured using a six-item scale, which asked participants how confident they were keeping fatigue, physical discomfort, pain, emotional distress, and other symptoms and health problems caused by cancer diagnosis and treatment from interfering with the things they want to do; they were also asked about their confidence doing different tasks and activities needed to manage their cancer diagnosis and treatment to reduce their need to see a doctor. Responses to these items ranged from Not at all confident to (1) to Totally confident (10). If respondents answered at least four of these items, the scale was calculated as the mean of the non-missing items. Higher scores represented greater confidence. An increase or positive change was desired.
- Health status: we asked respondents to rate their health on a scale of excellent (1) to poor (5). A low value on this scale indicates better health; a decrease or negative change from the base-line period to the final period for this variable.
- Health symptomatology:
  ○ Energy: we asked patients five questions about their level of energy: (1) Do you feel worn out?, (2) Did you have a lot of energy?, (3) Did you feel tired?, (4) Do you have enough energy to do the things you wanted to do?, and (5) Did you feel full of pep?. Responses to these items range from none of the time (0) to all of the time (5). If the respondent replied to at least three of these five items, the scale was calculated as the mean of the non-missing items with the two negatively worded items (1 and 3) reversed coded. A high score on this scale represents more energy. An increase or positive change for scale is desirable.
  ○ Pain, stress, and sleep problems: these three visual scales ranged from no problem (0) to very big problem (10). A high score on these scales represents more problems. A decrease or negative change on this scale was desired.
- Depression: participants completed the eight-item Personal Health Questionnaire Depression Scale (16). The items’ responses ranged from Not at all (0) to Nearly Everyday (3). Sum scores ranged from 0 to 24. Higher scores indicate more severe depression. A decrease or negative change in these items was desired.

### Statistical analysis

Pearson Chi Square and Fisher exact tests were used in Table 1 to compare the demographic characteristics of the intervention and control groups.

**Table 1:** Characteristics at baseline among study participants*, by treatment group (*n*, %).

Characteristic	Intervention (*n* = 169*)	Control (*n* = 89*)	*p*-Value
Age (years)
<50	33 (19.5)	19 (21.4)	0.93
50–64	81 (47.9)	41 (46.1)	
65+	55 (32.5)	29 (32.6)	
Sex
Male	38 (22.5)	9 (10.1)	0.01
Female	131 (77.5)	80 (89.9)	
Marital status
Married/partner	106 (62.7)	47 (52.8)	0.19
Single	62 (36.7)	42 (47.2)	
	1 (0.6)		
Hispanic ethnicity	13 (7.7)	6 (6.7)	0.78
Race
White	145 (85.8)	74 (83.2)	0.57
Black	14 (8.3)	5 (5.6)	0.43
Other**	11 (6.5)	11 (12.4)	0.11
Insurance
Medicaid	10 (5.9)	3 (3.4)	0.55
Medicare	83 (49.1)	31 (34.8)	0.03
HMO (Kaiser)	28 (16.6)	8 (9.0)	0.09
Private	63 (37.3)	34 (38.2)	0.88
VA/Other	4 (2.4)	2 (2.3)	1
None	6 (3.6)	3 (3.4)	1
Employment
Working	47 (27.8)	38 (42.7)	0.04
Not working	49 (29.0)	15 (16.9)	
Retired	49 (29.0)	25 (28.1)	
Other	11 (6.5)	2 (2.3)	
Missing	13 (7.7)	9 (10.1)	
Self-rated health			
Excellent	13 (7.7)	8 (9.0)	0.76
Very good	52 (30.8)	29 (32.6)	
Good	77 (45.6)	35 (39.3)	
Fair	25 (14.8)	15 (16.9)	
Poor	2 (1.2)	1 (1.1)	
Missing	0 (0.0)	1 (1.1)	
Years since diagnosis	18 (10.7)		
<1	73 (43.2)	7 (7.9)	0.87
1–3	44 (26.0)	41 (46.1)	
4–9	29 (17.2)	24 (27.0)	
10+	5 (3.0)	13 (14.6)	
Missing		4 (4.5)	
Cancer type			
Breast	66 (39.1)	66 (74.2)	<0.0001
Lymph./Hodgkins	27 (16.0)	4 (4.5)	0.01
Prostate	12 (7.1)	4 (4.5)	0.41
Colorectal	11 (6.5)	2 (2.3)	0.23
Endometrial/uterine	7 (4.1)	1 (1.1)	0.27
Ovary	9 (5.3)	0 (0.0)	0.03
Multiple myeloma	6 (3.6)	2 (2.3)	0.72
Lung	9 (5.3)	1 (1.1)	0.17
Leukemia	6 (3.6)	4 (4.5)	0.74
Melanoma	4 (2.4)	1 (1.1)	0.66
***Other	43 (25.4)	6 (6.7)	0.0003

*Includes persons diagnosed with cancer (excludes participating caregivers).

**Anyone who did check black or white including those who checked Asian, Native American, or other.

***Including cancer of the cervix, bladder, bone, brain, esophagus, kidney, liver, pancreas, thyroid, or other.

In order to determine if the outcome variables showed the change in the desired direction over time, we used repeated measures analysis with an unstructured variance covariance matrix. This method models the correlations between repeated observations from the same individual. It also utilizes available data for all participants, regardless of final measure completion allowing for a form of intent to treat analysis, which reduces potential drop-out bias. A case is only excluded if they did not answer a sufficient number of items on both the pre- and the post-test. If they have enough data for either time period, they were included in the sample. Data from participants were only excluded if they did not supply an adequate number of responses required for each instrument; if they had enough responses for either time point the data were included in the analysis. Each of the 10 outcomes described above served as dependent variables in models with no intercepts and a time period (baseline, final) by group assignment interaction as the independent variable. Parameters resulting from this model include an estimated mean for each group at each time period (17). Contrasts were estimated to determine change from baseline to final and differences between groups.

We conducted additional analyses to determine if the effect of the intervention was moderated by age. These models were similar to the models described above except a three-way interaction of age group (<65, 65+), treatment group (intervention vs. control), and time period since diagnosis replaced the two-way interaction. No interaction effect was observed, so the original analyses are presented.

Effect sizes were calculated using Cohen’s *d* (18), which is defined as the difference between two means divided by the pooled SD of the groups. Analysis was conducted using the Mixed Procedure of SAS 9.4. Unlike statistical significance, effect size is not dependent on sample size for interpretation. Effect size is a quantitative measure of the relative strength of the intervention whereby a larger absolute effect size value always indicates a stronger effect.

## Results

### Recruitment and randomization

**Figure 1:**
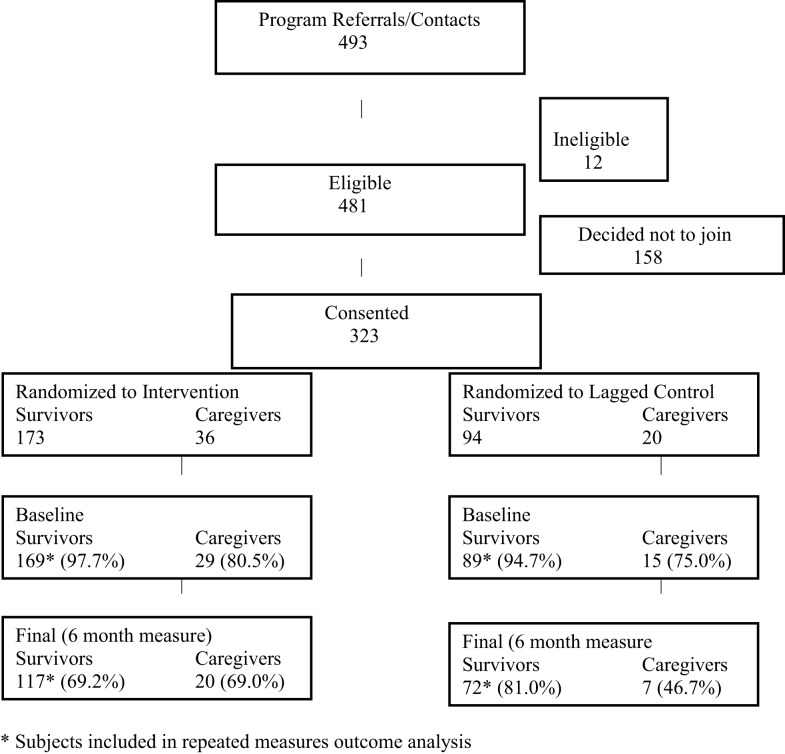
**Study participation**.

The activities described above resulted in 493 referrals (see Consort Diagram, Figure 1). Since this was an effectiveness study, the eligibility criteria were quite broad and only 12 of these individuals were ineligible (reasons included: did not receive treatment for cancer, over age 79, and still in treatment). A total of 158 subjects ultimately did not enroll as follows: did not show for first session to sign consent form (*n* = 37); not interested after learning more (*n* = 31); unreachable/voicemail left (*n* = 38); bad timing/inconvenient time/location (*n* = 34); in cancer treatment because cancer returned following initial outreach (*n* = 18). This resulted in a total of 323 eligible subjects enrolled, including 267 persons diagnosed with cancer and 56 caregivers/supporters. Randomization resulted in 169 survivors (and 29 of their caregivers) assigned to the intervention and 89 survivors (and 15 of their caregivers) assigned to the control. Only the survivors (not caregivers) in each group were utilized for the comparisons described in this paper (see below).

### Participation/completion

The average number of participants in each workshop was 8.2 ± 2.7; half the sessions had 7–9 participants (48.2%) with the remainder of workshops approximately evenly split between 5and 6 (29.6%) or 10 or greater participants (22.2%). The majority of participants (84%) completed four or more of the six sessions in each workshop (data not shown). Of the 169 persons diagnosed with cancer who were assigned to the intervention, 117 completed the final program measure (69.2%). A similarly high percentage of persons diagnosed with cancer and assigned to the control group (*n* = 89) completed the final measure (*n* = 72; 81.0%). Baseline characteristics of completers and non-completers were compared (data not shown) with only one factor (gender) significantly different between groups (more women in the intervention group); differences by intervention vs. control group in regard to completion were not observed. Baseline characteristics by group assignment are shown in Table 1; the groups were quite similar in accordance with major characteristics with the exception of gender and cancer type. Differences in insurance type and employment status were also present.

### Missing data/data entry errors

Five percent of surveys entered were checked at random for data entry errors; these checks were performed by study personnel who did not perform the initial data entry and demonstrated a detected error rate of <1%. A total of 448 surveys (258 baseline, 190 post-program) were collected. The number of surveys with complete data for specific outcomes ranged from 427 to 448, indicating that missing data within available surveys was minimal.

### Outcome measures

Baseline, final values measured at 6-month post-program, and change (%) values over time observed among participants in the intervention as compared to the control group are shown in Table 2. Statistically significant change over time among participants in the intervention was observed in the following outcomes: provider communication, depression, energy, sleep, and stress. Where change was observed in the controls, they were smaller among most outcomes.

**Table 2:** **Baseline, final, change, and % change between 6-month outcome measures, by intervention vs. control**.

	Intervention (*n* = 169)				Lagged control (*n* = 89)			
6-month outcome	Baseline value mean (SE)	Final value mean (SE)	Change mean (SE)	Change (%)	Base-line value mean (SE)	Final value mean (SE)	Change mean (SE)	Change (%)
Days active	3.0 (0.2)	3.0 (0.2)	0.1 (0.2)	1.7	2.9 (0.2)	2.8 (0.3)	−0.1 (0.3)	−4.3
Minutes active	143.4 (12.3)	192 (27.3)	48.7 (28.2)	34.0	124.4 (17.0)	155.1 (35.0)	30.6 (36.5)	24.6
Self-efficacy	70.2 (1.7)	72.4 (2.0)	2.3 (1.9)	3.2	73.6 (2.3)	77.7 (2.6)	4.0 (2.5)	5.5
Provider communication	3.1 (0.1)	3.7 (0.1)	0.5** (0.1)	16.7	3.5 (0.1)	3.8 (0.1)	0.3 (0.1)*	7.4
Depression	8.5 (0.4)	6.9 (0.5)	1.62** (0.5)	−19.1	7.8 (0.6)	7.2 (0.6)	−0.7 (0.6)	−8.5
Health status	2.7 (0.1)	2.7 (0.1)	0.0 (0.1)	0.4	2.7 (0.1)	2.6 (0.1)	−0.1 (0.1)	−2.2
Energy	2.2 (0.1)	2.5 (0.1)	0.3** (0.1)	13.8	2.1 (0.1)	2.4 (0.1)	0.3 (0.1)*	12.9
Pain	3.2 (0.2)	3.0 (0.2)	−0.3 (0.2)	−8.0	3.5 (0.3)	3.7 (0.3)	0.2 (0.3)	5.9
Sleep problems	5.3 (0.2)	4.0 (0.3)	−1.3** (0.2)	−24.9	5.6 (0.3)	4.9 (0.3)	−0.7 (0.3)*	−12.7
Stress problems	5.1 (0.2)	4.1 (0.2)	−1.0** (0.22)	−19.1	5.4 (0.3)	4.8 (0.3)	−0.6 (0.3)*	−10.3

*Statistically significant change between baseline and final measures; *p* <0.05.

**Statistically significant change between baseline and final measures; *p* < 0.001; repeated measures analysis.

### Effect sizes

Effect sizes calculated by Cohen’s *d* are shown in Table 3 for the intervention, control, and the difference in degree of change between the two groups. A beneficial effect was observed over time among participants in the intervention for many outcomes, consistent with the results in Table 2. For example, medium effect sizes (0.5–0.75) were shown for provider communication, depression, energy, sleep, and stress. In contrast, small effect sizes (0.16–0.35) or no effects were observed in the control for these same outcomes.

**Table 3:** **Effect sizes (Cohen’s *d*) in 6-month outcomes, by intervention vs. control and change between groups**.

Outcome	Effect size in intervention group (*n* = 169)	Effect size observed in control group (*n* = 89)	Effect size of difference in change between intervention and control
Days active	0.03	−0.05	0.06
Minutes active	*0.25	0.12	0.06
Self-efficacy	0.17	*0.23	0.08
Health status	0.02	−0.08	0.08
Provider communication	**0.75	*0.29	*0.23
Depression	**−0.50	*−0.16	*−0.18
Energy	**0.51	*0.35	0.03
Pain	−0.17	0.11	*−0.19
Sleep problems	**−0.72	*−0.31	*−0.20
Stress problems	**0.63	*−0.28	*−0.17

*Borderline/small effect (0.2).

**Medium effect (0.5).

When the effect size for the difference in change in the intervention group relative to the change in the control was evaluated, a small effect was observed in regard to provider communication (0.23), sleep (−0.20), and very small effect for stress-related problems (−0.17).

In addition to the results shown in Tables 2 and 3, we also examined the role of interaction between age, time since diagnosis, and outcomes of interest and did not detect possible effect modification (data not shown).

## Discussion

These outcome analyses of the adapted version of the Stanford CDSMP for cancer survivors indicate demonstrable beneficial effects in many outcomes (Tables 2 and 3); further, no outcomes worsened following participation in the intervention when we evaluated the group-level comparisons. The outcomes that improved with the CTS program (e.g., provider communication, depression, sleep problems, and stress-related problems; see Table 3) are particularly salient to the challenges faced by cancer survivors. For example, fragmented and poorly coordinated systems of care make provider communication an important skill for the cancer survivor. Sleep, depression, and pain are commonly reported symptoms as described in the previously cited IOM report, and it is notable that a non-medical, relatively inexpensive and brief educational intervention delivered in the community setting had positive impact on these common yet potentially serious issues.

Improvement over time between the baseline and 6-month measure was also observed in three domains among the controls, although generally to a lesser degree than those in the intervention (see Table 2). For example, we observed a 19.1% mean difference depression scores measured by the PHQ-8 over time in the intervention group in contrast to only 8.5% decline in the controls over time. Further, the difference over time was statistically significant in the intervention but not in the control group. This difference in the magnitude of effect over time observed in the intervention vs. control groups is illustrated by comparing effect size. Effect sizes take into account the size of measurement error in the data but do not rely on sample size or statistical significance for their interpretation; therefore, they are meaningful when evaluating the relative impact of an intervention. In the case of depression, for example, the effect size in the intervention group over time was a medium/large effect (−0.50) vs. a small effect (−0.16) in controls (Table 3). Similarly, striking differences in effect size among the intervention vs. control groups were observed for pain (−0.17 vs. 0.11), suggesting a net small/medium benefit since the trend in the controls was to worsen over time. Although sample size does not directly impact the calculation of effect size, the finding of effect size differences is considered meaningful when observed in larger, well-designed studies such as reported here.

The heterogeneous nature of our study population in this intentionally pragmatic design allows us to estimate the benefits of the program in the real world by examining effect sizes (i.e., effectiveness). However, it also dampens the ability to detect statistically significant differences because by design, it does not use carefully constructed homogeneous study populations to minimize variation as in efficacy trials. The ability to demonstrate statistical significance in an effectiveness evaluation is strongly influenced by the number of persons in relevant subgroups where the intervention may be more or less efficacious; however, these subgroups are not necessarily known to the researchers or able to be detected in the real-world setting of the evaluation. The fact that we that we did not see statistically significant difference between the change over time in the intervention vs. control is likely a consequence of the heterogeneous nature of our real-world study population; but the difference in effect sizes represents the impact of the intervention by measuring the magnitude of this difference observed over time in the two groups and highlights the external validity of our findings.

Other studies of the CDSMP have similarly evaluated effect size to evaluate the impact of the program. A 2008 Cochrane Collaboration review (11) of self-management education interventions demonstrated effect sizes observed in multiple reports of other populations similar to or smaller than those observed in the current study. For example, of the 17 randomized trials of lay-led self-management programs in this review demonstrated effect sizes for pain of 0.10 (current study 0.11) and depression of 0.16 (current study 0.18).

Although we did not observe an effect with self-efficacy as observed in other trials (ranging from 0.30 to 0.40), we did observe an improvement in provider communication (0.23). Cancer survivors in the post-treatment period neither have the frequency nor regularity of health system interaction as with other chronic conditions such as asthma and diabetes and therefore may not have had ample opportunity to use their self-management skills, which could be the cause of the neutral scores on this domain. However, the improvement observed in provider communication is a related and similarly important skill for this population. Provider communication is a necessary component of the Chronic Care Model, which promotes collaboration between patients and providers in partnership to achieve improved outcomes (5). This is especially important in survivors who may experience both late and long-term side effects from treatment that can change over time, and may require ongoing vigilance and care.

Another observation from our study could be explored in future research is our observation of improvement over time in the control group, which although was to a lesser degree, was statistically significant in three constructs (energy, sleep, and stress). Other researchers have suggested that positive adjustment or post-traumatic growth over time following a stressful event such as cancer can occur (19–21). Thus, one possible explanation for this finding is that survivors have accepted a “new normal” and therefore the increase over a time is a reflection of this perception. Additionally, there may be some endogenous aspects to a survivor’s improvement that could be capitalized upon in future iterations of the program.

Limitations of this study are that we may not have quantitatively measured all the outcomes of relevance in this population. For example, we did not directly measure social support or the unique benefits among caregivers such as family communication. Additionally, while the majority of respondents completed the final measure, we were unable to measure final outcomes in all respondents. However, we utilized repeated measures analysis to utilize data from all respondents regardless of completion to minimize this potential source of bias. Additionally, we chose to include survivors in the post-treatment stage only to support the unmet need for transition support. Although it is reasonable to expect that similar benefits would be observed in survivors at other points in the continuum, additional evaluations with survivors at other time points should be conducted.

A recent review of 16 self-management programs that have been utilized with a variety of cancer survivor populations promotes the use of the Chronic Care Model and particularly support for self-management in addressing needs across the continuum from diagnosis to survivorship (22). Aspects of self-management highlighted in this review as beneficial for survivors are also highly visible “active ingredients” in the CTS program and include: goal setting, realistic action plans, partnering with providers, and identifying aspects of health and healthcare that patients can self-manage with confidence. Although the attention to self-management interventions in this population is increasing, this review concludes that there is an urgent need for the translation of these interventions into practice, particularly in the post-treatment period. The authors suggest that interventions at this point in time can be especially helpful in easing transition to less regular contact with oncologists and dealing with the psychosocial and functional challenges into survivorship.

Contemporary views of effectiveness have evolved to suggest that it is influenced not only by efficacy, but reach of the program as well as implementation with fidelity (23). Our enrollment of over 300 cancer survivors and caregivers to this effectiveness study and the diversity of the study population according to cancer type, time since diagnosis, age, and other characteristics as shown in Table 1 suggests that this program can reach and appeal to the general cancer population. By partnering with a community agency with state-wide reach for delivery of the original CDSMP Program with certified facilitators and extensive experience in delivering the program, we were further able to deliver the new adapted version in keeping with the principles of original program method. When taken in sum, these outcome and implementation data demonstrate that survivors who participate in the CTS program experience a small but measurable net gain over time in important survivorship domains in comparison to those who receive no intervention, and that the program can fill an important gap in meeting the ongoing need for management of post-diagnosis issues in this growing segment of the U.S. population.

The implementation of self-management support is particularly challenging in the cancer environment for a number of reasons including the use of multiple specialty care providers from diagnoses through to treatment and survivorship, lack of an evidence base to guide follow-up surveillance and decision making, complex late and long-term side effects requiring detailed patient history and records, and limited oncology system capacity. While the delivery of self-management programs to date has been driven by innovations in primary care, a recent emerging trend in support of needed system and policy change for cancer survivors is the establishment of patient-centered medical homes (PCMH) in the oncology setting (24). Future research is needed to support policy change to ensure that patients receive self-management support that is tailored to their cancer needs across oncology and a variety of other settings, driven by patient needs and preferences. Additional research is also needed to understand which outcomes are most relevant in this population toward demonstrating cost-effectiveness that can inform needed system and policy change.

Decreased emergency room visits and hospitalizations are of relevance to cost in other chronic illness populations (14), but outcomes such as overuse of care/screening may be even more important for cancer survivors. Patterns of care outcomes are difficult to track in with multiple payor systems, but policy changes to support the collection and analysis where possible in Medicaid/Medicare or other single-payor systems should be pursued to further evaluate outcomes from self-management support for cancer survivors. The CTS has enormous potential to be widely disseminated by tapping into existing channels in the community and among providers that have already been established with the CDSMP Program; however, the successful implementation of self-management interventions such as the CTS is reliant upon buy-in by oncology providers, survivors, and the healthcare system to recognize benefits such as those observed in the current report. As evidence continues to mount on the effectiveness of the CDSMP in other chronic disease populations (25, 26), and models of survivorship care continue to develop, policy and system support for self-management as a vital and viable component in successful transition to survivorship is needed.

## Conflict of Interest Statement

Dr. Lorig receives royalties from Bull Publishing Company (Boulder, CO, USA) for the book that is included in the CDSMP delivery. The research was conducted in the absence of any commercial or financial relationships that could be construed as a potential conflict of interest among other authors.

This paper is included in the Research Topic, “Evidence-Based Programming for Older Adults.” This Research Topic received partial funding from multiple government and private organizations/agencies; however, the views, findings, and conclusions in these articles are those of the authors and do not necessarily represent the official position of these organizations/agencies. All papers published in the Research Topic received peer review from members of the Frontiers in Public Health (Public Health Education and Promotion section) panel of Review Editors. Because this Research Topic represents work closely associated with a nationwide evidence-based movement in the US, many of the authors and/or Review Editors may have worked together previously in some fashion. Review Editors were purposively selected based on their expertise with evaluation and/or evidence-based programming for older adults. Review Editors were independent of named authors on any given article published in this volume.

## Footnotes

^1^http://patienteducation.stanford.edu/programs/cdsmp.html

^2^http://patienteducation.stanford.edu/research
